# 

**Published:** 1892-09-17

**Authors:** 


**Notices of Births, Deaths, and Marriages,** are inserted in this column at a charge of 2s. 6d. for an announcement not exceeding four lines.

### NOTICE TO CORRESPONDENTS.

**All MS., Letters, Books for Review, and other matters intended for the Editor should be addressed** The Editor, The Lodge, Porchester Square, London, W.

**The Editor cannot undertake to return rejected M.S., even when accompanied by stamped directed envelope.**

### Notice to Advertisers and Subscribers.

**All Advertisements, Orders for Copies of The Hospital, and Business Communications should be addressed to The Publisher, *not to the Editor*, The Hospital, 140, Strand, W.C.**

**The Cost of Subscription, post free, is as follows:—Per week, 2½d.; per quarter, 2s. 6d.; per half-year, 5s.; per annum, 8s. 6d. Foreign:—Per annum, 10s. 8d.; payable, in all cases, in advance.**

### "Burdett's Hospital Annual," 1891—1892.

Third year of publication. 600 pp. Boards, gilt lettering. A most complete guide to British and Colonial Hospitals, Dispensaries, Nursing and Convalescent Institutions, and Asylums. Price 3s. 6d. Published by The Scientific Press (Limited), 140, Strand, W.C.

**NOTICE.—The Index for Vol. XI. (ending March, 1892) is on sale at the Office of this Journal, price 2½d., post free.**

### Scraps and Gleanings.

As the result of Hospital Saturday the Malvern Rural Hospital and Dispensary benefited to the amount of £46 15s. 2d.

A shark measuring nearly 8 feet has been captured at Stoke, below Rochester. An exciting hunt took place before the shark was finally driven into a shallow creek of the Medway, and there killed.

The sum of £5,400 has already been received towards a memorial to the late Cardinal Manning, in the shape of a refuge, "under Catholic management, for the homeless poor, without distinction of creed."

The visit of the Czar and Empress to the cholera hospitals and their kindness to the patients, has, says a St. Petersburg telegram, evoked an outburst of patriotic eulogy from all classes of the inhabitants.

At a meeting of the Mansion House Council, Mr. Alexander presiding, the receipt of a grant of £5,000 from the charity legacy of the late Mr. Richard Berridge, for the Dwellings of the Poor was announced.

The following legacies to hospitals are announced: £200 to the West London Hospital, Hammersmith, from Mrs. Dalgairns, of Clifton, and £50 to the Potters Bar Cottage Hospital, from the Dowager Countess of Strafford.

The late Mr. Joseph Hobson, proprietor of the Theatre Royal, Leeds, has left £100 a year for ten years to the Leeds Infirmary, and £50 a year for ten years to the Leeds Dispensary, and smaller legacies to other charities.

Despite the inclemency of the weather the sum of £123 11s. 9d. was collected in Watford on Hospital Saturday, which is to be divided between the Watford Cottage Hospital, the Herts Convalescent Home, and the Nurse Fund.

On Hospital Saturday at Ilfracombe fruit and flowers were sold for the benefit of the Tyrrell Cottage Hospital. In the evening there was a grand concert, which was largely attended. The sum collected amounted to about £[illegible]00.

The friendly societies of Tonbridge and District recently held their annual Sunday parade in aid of the Tunbridge Wells Eye and Ear Hospitals. One of the chief features of the proceedings was a collection box carried by Mr. Bates' hospital dog "Help."

Mr. J. Bradley, the Secretary of the Stockton Infirmary, read a gratifying report of its progress during the last year. He stated that altogether 2,369 patients were treated during the past twelve months, being 243 in excess of the previous year.

The Dartmouth Friendly Societies mustered in force at Warfleet for the annual hospital parade. The amount cleared was £24 6[illegible]. 2½d. This showed a lamentable falling off of over £12 from the last year's collection, which reached the total of £36 8s. 8d.

The Exmouth Local Board have taken prompt measures to prevent the establishment of a fever or cholera hospital in or near the town, for cases brought into the port by ships trading with foreign countries. It will therefore be necessary for them to provide a floating hospital.

It is hoped that before this time next year the new Poplar Hospital which is so sorely needed will be completed. Of the £17,000 required, £14,000 has already been promised, and the committee trust others will help them to raise the required sum, so that the hospital may be opened free of debt.

Towards the building of the National Hospital for Consumption in Ireland £10,000 has now been promised. Miss Florence Wynne has herself collected subscriptions amounting to £1,9[illegible]0, and the Countess of Zetland has from the first been most indefatigable in promoting and supporting the movement.

The members of the Hospital Saturday Committee of the Wolverhampton General Hospital have presented Mr. T. V. Jackson with a carriage clock and a photograph of the members of his ambulance class, in acknowledgment of the invariable courtesy and kindness which he had always shown them.

A most desperate fight took place last week at Luton between a monkey and a serpent belonging to Sanger's Circus. Both animals fought for a considerable time in spite of every effort made by the keepers to separate them. At last it was found necessary to kill the serpent in order to save the monkey.

The death is recorded of Dr. J. E. Morgan, of Manchester. He had a share in founding the Manchester Nurse Training Institution, a Professor of Medicine at Owens College, and a member of the Royal College of Physicians. He was the author of several medical works, especially "The Danger of Deterioration of Race."

The procession at St. Albans this year in aid of the Cottage Hospital was an unusually attractive one. There were emblematical cars and characters in costume, among them one filled with young girls dressed as hospital nurses, another representing the village blacksmith, chestnut tree and all complete. A bright fire was burning and the brawny-armed one was busily engaged in forging shoes, &c.

The foundation-stone of the Chorley Cottage Hospital was laid by the wife of the donor, Mrs. H. Rawcliffe. The site was given by the Rev. J. J. Levion, and adjoins the dispensary. The building will be two storeys high. There will be four wards providing accommodation for twelve beds and a convalescent room. A balcony is to extend along the south front of the building.

The "Mary Hewitson" Cottage Hospital, at Keswick, built by Mr. H. Hewitson, in memory of his sister, has just been opened. It is built in the Queen Anne style, and provides accommodation for ten beds, all on the ground floor. There is a large ward 22 ft. by 20 ft., and a smaller one 14 ft. by 12 ft. on each side of the main entrance, and separated by the nurses' room. There are four beds in each of the larger and one in the smaller wards.

Five years ago three boys were bitten by a strange dog. A few days afterwards all three were sent to Paris and underwent M. Pasteur's treatment. One boy, five weeks afterwards, died of hydrophobia in Huddersfield Infirmary. The other two enjoyed fairly good health until a week ago, when one of them complained of pains in the arms and body, showed an aversion to liquid, and died after a few days' illness. At the inquest a verdict was returned of "Death from hydrophobia, caused by the bite of a dog five years ago."

### THE STUDENT OF MEDICINE.

[All communications for this Supplement should be addressed to THE EDITOR of THE HOSPITAL, at 140, Strand, with the words, "Students' Column," written in the left hand top corner of the envelope.]

### APPOINTMENTS.

Ashworth, J. Henry, M.D., M.R.C.P.Edin., reappointed Honorary Medical Officer to the Halstead Cottage Hospital. Atkey, P. J., L.R.C.P., M.R.C.S., appointed House Surgeon to St. Thomas's Hospital. Banks, A., L.R.C.P., M.R.C.S., appointed House Surgeon to St. Thomas's Hospital. Bowring, W. A., L.R.C.P., M.R.C.S. (extension), appointed Resident House Physician to St. Thomas's Hospital. Bromley, John B., M.R.C.S., L.S.A., reappointed Honorary Medical Officer to the Halstead Cottage Hospital. Bulman, F., M.B., B.S.Durh., appointed Medical Officer for the Workhouse of the Newport (Mon.) Union. Burden, H., L.R.C.P., M.R.C.S., appointed House Surgeon to St. Thomas's Hospital. Caley, Henry Albert, M.B.Lond., L.R.C.P., M.R.C.S., appointed Medical Registrar to St. Mary's Hospital, W. Dalzell, A., L.R.C.P., M.R.C.S. (extension), appointed Clinical Assistant to the Department for Diseases of the Throat at St. Thomas's Hospital. Dorman, U. R. P., M.A., M.B., B.C.Cantab., L.R.C.P., M.R.C.S. (extension), appointed Clinical Assistant to the Department for Diseases of the Throat at St. Thomas's Hospital. Fisher, J. H., L.R.C.P., M.R.C.S., appointed House Surgeon to St. Thomas's Hospital. Fooks, W. P., M.A., M.B., B.C.Cantab., L.R.C.P., M.R.C.S. (extension), appointed Non-Resident House Physician to St. Thomas's Hospital. Frederick, H. J., L.S.A., appointed Clinical Assistant in the Department for Diseases of the Ear at St. Thomas's Hospital. Fullston, Andrew, M.B., B.Ch., appointed House Surgeon to the West Kent General Hospital, Maidstone. Gunn, Robert Marcus, M.A., F.R.C.S., appointed Honorary Ophthalmic Surgeon to the Cheyne Hospital for Sick and Incurable Children, Chelsea. Haydon, T. H., B.A., M.B., B.C.Cantab., L.R.C.P., M.R.C.S., appointed Senior Obstetric House Physician to St. Thomas's Hospital. Law, R. R., B.A., M.B., B.C.Cantab., appointed Assistant House Surgeon to St. Thomas's Hospital. Lovell, C.P., M.A., M.B., B.Ch.Oxon, L.R.C.P., M.R.C.S., L.S.A. (extension), appointed Clinical Assistant in the Department for Diseases of the Skin in St. Thomas's Hospital. Montgomery, W. P., B.A.Oxon., M.R.C.S., L.R.C.P., appointed Senior House Surgeon to the Ancoats Hospital. Norgate, R. H., M.R.C.S.Eng., L.R.C.P.Lond., appointed Senior Assistant Medical Officer to the Kent County Asylum, Barming Heath. Price, A. E., L.R.C.P, M.R.C.S., appointed Clinical Assistant in the Department for Diseases of the Ear at St. Thomas's Hospital. Purvis, W. P., B.Sc.Lond., L.R.C.P., M.R.C.S., appointed Clinical Assistant in the Department for Diseases of the Skin at St. Thomas's Hospital. Ryall, Charles, L.R.C.S.Edin., L.R.C.P.Edin., L.F.P. and S.Glas., appointed Resident Medical Officer to the Victoria Hospital, Lewes. Simpson, H., B.A., M.B., B.C.Cantab., L.R.C.P., M.R.C.S., appointed Assistant House Surgeon to St. Thomas's Hospital. Skottowe, A. J. F., M.D., C.M.Glas., appointed Hospital Surgeon, Medical Officer for the Burgh, and Police Surgeon, Helensburgh. Wallace, C. S., L.R.C.P., M.R.C.S., appointed Junior Obstetric House Physician to St. Thomas's Hospital. Wallace, F. G., M.A., M.B., B.C.Cantab., L.R.C.P., M.R.C.S. (extension), appointed Non-Resident House Physician to St. Thomas's Hospital. Watkins-Pitchford, W., L.R.C.P., M.R.C.S., appointed Resident House Physician to St. Thomas's Hospital.

### THE STUDENT OF MEDICINE—(*continued*).

### VACANCIES.

The following vacancies are announced this week, the amount of salary in each case being given after the name of the institution:

#### Resident Medical Officers.

County Asylum, Prestwich, Manchester, Assistant Medical Officer—Salary £100 per annum, with a prospect of an increase of £25 at the end of first year, and of £25 at the end of second year.

General Hospital, Birmingham, House Physician—Salary £70 per annum.

Lancashire County Asylum, Assistant Medical Officer—Salary £200 per annum.

Parish of St. Leonard, Shoreditch, Clinical Assistant for the Infirmary, Hoxton Street, N.—Salary £40 per annum, with rations, furnished apartments, and washing.

Scarborough Hospital and Dispensary, House Surgeon—Salary £80 per annum.

Sussex County Hospital, Brighton, House Surgeon, doubly qualified, unmarried, and under 30 years of age—Salary £120, rising to £140 per annum, with board and washing.

Worcester County and City Lunatic Asylum, Third Assistant Medical Officer—Salary £100 per annum.

#### Non-Resident Medical Officers.

General Hospital, Birmingham, Pathologist—Salary £120 per annum.

General Hospital, Birmingham, Surgical Casualty Officer—Salary £70 per annum.

Liverpool Hospital for Cancer and Skin Diseases, Honorary Assistant Surgeon.

Sunderland Corporation, Medical Officer of Health for the District of the Borough and Port—Salary £500 per annum as Medical Officer of the Borough, £20 for the like office of the Port, and £5 as Public Analyst.

University of Aberdeen, Six Examiners for Graduation in Medicine. Grant of £30 per annum each.

### PASS LISTS.

#### Society of Apothecaries of London.

AUGUST, 1892.

The following candidates passed in:

SURGERY.—W. D. Akers, St. Mary's Hospital; D. Berne, Royal Free Hospital; F. G. Dorey, Yorkshire College, Leeds; E. G. Firth, Yorkshire College, Leeds; W. Fowler, Durham; E. E. Francis, St. Bartholomew's Hospital; W. Handcock, Yorkshire College, Leeds; B. H. F. Leumann, St. Bartholomew's Hospital; W. S. Mercer, Charing Cross Hospital, W. H. F. Noble, London Hospital; A. V. Peatling, St. Thomas's Hospital; B. Saul, Charing Cross Hospital; D. L. Thomas, London Hospital; W. Thomas, Yorkshire College, Leeds; A. E. Wilson, St. Mary's Hospital.

MEDICINE, FORENSIC MEDICINE, AND MIDWIFERY.—R. S. Bernard, King's College; F. Dove, London Hospital; E. G. Firth, Yorkshire College, Leeds; V. P. Foote, Charing Cross Hospital; E. E. Francis, St. Bartholomew's Hospital; R. T. Gilmore, St. Mary's Hospital; J. L. Iredale, Yorkshire College, Leeds; J. Joule, London Hospital; H. L. A. Keller, St. Thomas's Hospital; M. C. Langford, London Hospital; B. H. F. Leumann, St. Bartholomew's Hospital; R. E. Nichols, St. Mary's Hospital; F. J. P. Smith, St. Thomas's Hospital; S. Smith, Middlesex Hospital.

MEDICINE AND FORENSIC MEDICINE.—J. E. Bailey, University College; W. Handcock, Yorkshire College, Leeds; J. J. Powell, St. Thomas's Hospital, and Cambridge; D. F. Roberts, Guy's Hospital; J. D. Willis, Manchester.

FORENSIC MEDICINE AND MIDWIFERY.—E. Howgate, Yorkshire College, Leeds; W. S. Newton, London Hospital; J. W. Roberts, Liverpool; W. H. Waddington, Owens College, Manchester.

FORENSIC MEDICINE.—J. B. Bate, Bristol; A. A. Macfarlane, St. Bartholomew's Hospital; A. W. Taylor, London Hospital; J. Wood, St. Thomas's Hospital.

Messrs. Akers, Bernard, Dove, Firth, Francis, Gilmour, Keller, Langford, Leumann, Macfarlane, Nichols, Noble, F. J. P. Smith, L. L. Thomas, and Waddington, were granted the diploma of the Society entitling them to practise medicine, surgery, and midwifery.

#### British Medical Service.

The following is a list of the successful candidates for Commissions in the Medical Staff of Her Majesty's Army at a recent examination in London:—

| | Marks. | | Marks. |
|---|---|---|---|
| C. E. Pollock | 2,815 | J. H. Farmer | 2,365 |
| F. M. Mangan | 2,470 | F. R. Buswell | 2,310 |
| J. H. Rivers | 2,470 | H. A. Berryman | 2,295 |
| W. J. Taylor | 2,450 | F. A. Symons | 2,285 |
| B. W. Longhurst | 2,385 | C. J. Samman | 2,235 |

